# Enhancing Solid Solution Strengthening of TiZrNb Alloys via W and Cr Alloying: First-Principles Insights into Mechanical Properties

**DOI:** 10.3390/ma19061069

**Published:** 2026-03-11

**Authors:** Zhichao Sun, Gaoyuan Ma, Qingshun Guo, Rongjiang Ou, Lei Guo, Cheng Ji, Zheng Zhang, Li Li, Chuanting Wang, Yong He

**Affiliations:** 1 School of Mechanical Engineering, Nanjing University of Science and Technology, Nanjing 210094, China; 2 Beijing Special Electromechanical Research Institute, Beijing 100081, China

**Keywords:** first principles, high entropy alloys, mechanical property, solution strengthening

## Abstract

This work investigated the effects of varying tungsten (W) and chromium (Cr) contents on the lattice constant, elastic properties, yield strength, and electronic structure of TiZrNb alloys via first principles and the Special Quasi-Random Structure (SQS). A modified Senkov approach, considering the local atomic environment to estimate yield strength was suggested. Analysis indicated that W and Cr decrease the lattice constant of the TiZrNb alloy. W could improve the elastic modulus of material and solid solution strengthening effect, but Cr only enhanced the bulk modulus at high levels. The alloying of W and Cr was not beneficial for enhancing the plasticity. Cr was more significant in damaging mechanical isotropy. The modified Senkov approach improved the estimation accuracy of yield strength. Electronic property analysis indicated that W and Cr could lower the Fermi level to enhance the stability of the phase. Their covalent interactions helped to enhance strength. At present, the accuracy of the theoretical predictions has improved, relative to the experimental values. This work will provide guidance for the design and optimization of TiZrNb-based alloys.

## 1. Introduction

Refractory high entropy alloys (RHEAs) are one of the main branches within the high entropy alloys (HEAs). This type of alloy is composed of high-melting-point metals and other elements serving as modifiers [1]. The change in the type and proportion of elements is one of the primary approaches for controlling the mechanical properties of alloys. Through extensive experimental attempts, scholars have discovered that adding HCP or FCC metals can improve the plasticity of RHEAs [2]. Therefore, from the initial two equiatomic alloys, MoNbTaW and MoNbTaVW [3], RHEAs have developed into numerous systems over the years. The frequently studied systems include MoNbTa, TiNbMo, TiZrHf, and TiZrNb, etc. [4]. Within these systems, a number of subsystems exhibit outstanding mechanical properties. Therefore, the origins of the mechanical properties in HEAs have also attracted attention.

Compared to conventional alloys, HEAs exhibit significant lattice distortion effects; hence, their mechanical properties primarily depend on solid solution strengthening [5]. The TiZrNb system is one of the more deeply researched systems. Not only have they developed sub-series exhibiting outstanding mechanical properties, but the solid solution strengthening effect in certain alloys can contribute over 80% of the strength [6]. Due to the relatively small mismatch in atomic radii and shear modulus between Ti, Zr, and Nb, the solid solution strengthening effect of the base TiZrNb alloy is limited. To enhance the solid solution strengthening effect, it is necessary to incorporate elements exhibiting significant differences in atomic radius or shear modulus compared to the matrix elements. This has led to the development of alloys such as TiZrNbTaMo, TiZrNbMoV, TiZrNbHfMo, and TiZrNbHf [7,8,9]. In the experimental research, the researchers have discovered a large number of alloy compositions with excellent mechanical properties by adjusting the composition and heat treatment process. For example, TiZrNbV_x_ [10], TiZrNbTa_x_ [11], Ti_3_Zr_1.5_NbV_x_ [12], (Ti_1.5_V)_x_(ZrNb) [13], Ti_x_ZrNbV [14] and (TiZr)_x_(HfNb)_100−x_ [15], etc. In some of these alloys, a degree of tensile plasticity is also exhibited at room temperature. In the theoretical research, Mashroor S. Nitol [16] developed a modified embedded atom method for TiZrNbTaV, which facilitates molecular dynamics studies of related alloys. Liao [17] investigated the effects of vanadium on the thermodynamic and mechanical properties of TiZrNb, using first-principles calculations and the virtual crystal approximation (VCA). Yang [18] employed the coherent potential approximation (CPA) to analyze the mechanical properties of the TiZrNbM (M = Hf, Ta, W). Wu [19] reported the mechanical properties of TiZrNbTaX alloys using SQS and VCA methods, noting that TiZrNbTaRe may exhibit higher strength compared to other elemental dopants. Zhang et al. [20] investigated the influence of chemical short-range ordering in TiZrHfNb on its mechanical properties. It is obvious that these alloys have already been extensively reported in both experimental and theoretical computational studies. It is noteworthy that, in these elements with a high solid solution strengthening capability, research on tungsten (W) and chromium (Cr) remains rare. However, W and Cr are highly suitable candidate solid solution elements in terms of atomic radius and shear modulus. Recently, Huang et al. [21] investigated the mechanical properties of TiZrNbW under different strain rates, discovering that tungsten enhances the strength of TiZrNb. At present, the influence of W and Cr on the mechanical properties of TiZrNb alloys remains predominantly experimental, with theoretical research being relatively poor.

Therefore, the SQS method was adopted to analyze the effects of W and Cr on the lattice constant, elastic modulus, and plasticity of the TiZrNb alloy. The influence of alloying on mechanical properties was revealed through the electronic density of states and electron difference density. Finally, this work adopted the Senkov approach, which considers the local atomic environment to estimate the strength of TiZrNbX (X = W, Cr) alloys. This work is expected to provide theoretical support for the design of RHEAs, thereby accelerating the discovery and application of innovative RHEA materials.

## 2. Method

TiZrNbW and TiZrNbCr were included in this work. The TiZrNbW_x_ and TiZrNbCr_x_ (x = 0.1, 0.25 and 0.4) crystal models were constructed. The crystal models of RHEAs were generated by adopting the Special Quasi-Random Structure (SQS). For the SQS, the Alloy Theoretic Automated Toolkit software (ATAT, Version 3.36) package was applied to create numerous 2 × 2 × 5 supercells [22]. This work was implemented using the Cambridge Sequential Total Energy Package (CASTEP, Version 2023) for first principles calculations [23]. Crystal structure relaxation used the Broyden–Fletcher–Goldfarb–Shanno method [24]. The structural relaxation convergence criteria were set as follows: the total energy changed was set to 10^−5^ eV, the maximum internal stress was 0.05 GPa, the maximum displacement value was set to 0.001 Å, and the convergence value of the maximum force between atoms was 0.03 eV/Å. The exchange-correlation functional was described by the GGA-PBE functional [25]. The electron–ion interactions were treated using an ultrasoft pseudopotential. Convergence calculations were performed using a self-consistent iterative method, with the convergence criterion set at 1.0 × 10^−6^ eV/atom. The cutoff energy was selected at 420 eV, and a 7 × 7 × 2 Monkhorst–Pack grid was set up for sampling the Brillouin zone. The local atomic analysis employed in this study was implemented via a Perl script.

## 3. Results and Discussion

### 3.1. Lattice Constant and Phase Structure

Since atoms deviate from their theoretical positions following structural relaxation, the lattice constant cannot be directly calculated. Therefore, the equivalent volume method ($a={\left(2V_{SQS}/n\right)}^{\frac{1}{3}}$) was used to indirectly calculate the lattice constant. Here, V and n are the volume and number of atoms in the crystal structure, respectively. Table 1 shows the lattice constants of the TiZrNbX alloy. The atomic radii of W ($r_{W}\;=\;136.7\;pm$) and Cr ($r_{Cr}\;=\;124.9\;pm$) are smaller than those of Ti, Zr, and Nb. Therefore, the lattice constant of the alloy decreased as their content increased. Due to the smaller radius of Cr, the lattice constant of the alloy decreased at a faster rate. Furthermore, this work calculated the average lattice constants of TiZrNbW and TiZrNbCr, based on Vegard’s law ($a_{Vegard}=\sum c_{i}a_{i}$). This trend was consistent with the values obtained from SQS, with more detailed findings indicating that for TiZrNbW, $a_{Vegard}$ was greater than $a_{SQS}$, whereas for TiZrNbCr, $a_{Vegard}$ was less than $a_{DFT}$.

**Figure 1:**
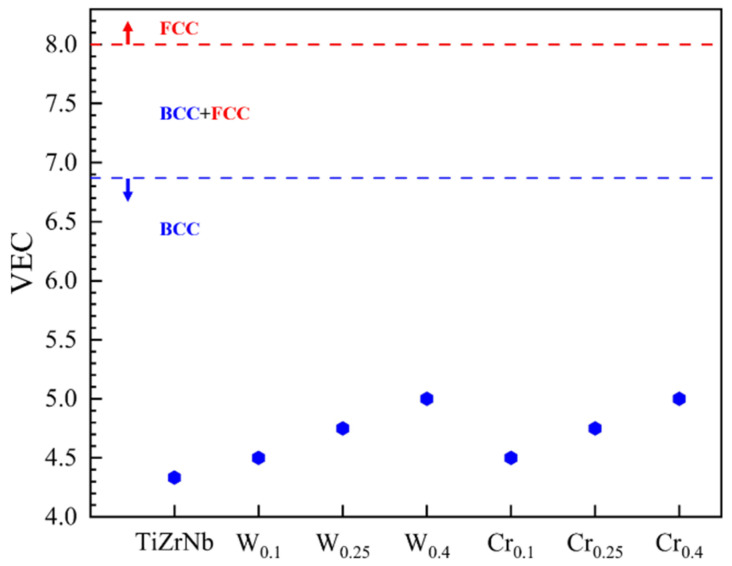
Valence electron concentration in TiZrNbX alloy.

Phase structure has an important influence on the properties of alloys. Phase structure can be adjusted by varying chemical composition and physical fields [27]. The valence electron rule is a simple and accurate empirical method for determining the phase structure. In HEAs, the BCC usually forms when the valence electron concentration (VEC) < 6.87 [28]. The VEC of the TiZrNbX alloy is shown in Figure 1. The VEC of all HEAs was less than 6.87, demonstrating that alloys could form a stable BCC structure. Although the VEC can determine the formation of the BCC structure, it is not able to predict the existence of other phases. Notably, the binary mixture enthalpy between the W or Cr and the matrix elements Ti, Zr, and Nb considered in this work is relatively low [29]. Therefore, the Laves phases are easily generated during the melting and cooling processes of alloys. In previous studies by our team, it was found that adjusting the W-Zr composition leads to the formation of W_2_Zr with varying sizes and distributions [30,31]. Xing et al. [32] found that Ti doping in WZr could effectively suppress the formation of the W_2_Zr, thereby indicating that alloying can theoretically control the formation of intermediate phases. Cr_2_Zr was also observed in TiZrNbCr during the experiments [33]. On the other hand, the addition of high-melting-point W will further increase segregation and form dendritic. Therefore, it is difficult to achieve the ideal phase structure by melting alone. Currently, it is still necessary to optimize the phase structure of such alloys via physical field regulation.

### 3.2. Elasticity Constant

In cubic crystals, the high symmetry reduces the original twenty-one elastic constants to three: $C_{11}$, $C_{12}$, and $C_{44}$. The Born’s stability criterion can be established according to the following equation [34]:

(1) $$\left\{\begin{array}{l} C_{44}>0\\ C_{11}>\left|C_{12}\right|\\ C_{11}+2C_{12}>0 \end{array}\right.$$

The elastic constants and Cauchy stresses for TiZrNbW, TiZrNbCr and TiZrNb with varying compositions are shown in Table 2. The related elastic constants for the TiZrNb alloy were obtained from the average values reported in previous studies. All TiZrNb RHEAs satisfied Equation (1), indicating the mechanical stability of structure. The elastic constants C_11_ and C_44_ measure the compressive and shear resistance of the material, respectively. It could be shown that pure metals exhibited high values for C_11_ and C_44_, indicating excellent compressive and shear resistance. Furthermore, although alloying enhanced the compressive strength of the TiZrNb, its shear resistance exhibited elemental dependence. In detail, W improved the shear resistance and gradually increased with the increase in compositions. However, the weakening effect of Cr on the shear capacity became increasingly significant.

The mechanical properties of materials can be predicted by elastic constants. Cauchy stress describes the bonding behavior of materials and reflects their ductility. A material displays metallic properties and good ductility if the Cauchy stress is positive. Conversely, when the Cauchy stress is negative, the material will exhibit covalent properties and consequently exhibit brittleness [35]. As shown in Table 2, the Cauchy stress of all alloys is positive, indicating that they exhibit good ductility. However, when W and Cr are doped, the Cauchy stress of the alloys first decreases and then increases. Thus, alloying reduces the ductility of the alloys to a certain extent.

**Table 2:** Single crystal elastic constants and Cauchy pressures of TiZrNb and alloying elements.

Alloy	$C_{11}$	$C_{12}$	$C_{44}$	$C_{p}$
W	510	201	143	58
Cr	499	139	102	37
TiZrNb [26,36,37]	146.7	102.0	29.4	72.6
TiZrNbW_0.1_	165.2	99.6	32.6	67.0
TiZrNbW	194.8	117.2	34.5	82.7
TiZrNbW_0.4_	240.9	130.3	43.6	86.7
TiZrNbCr_0.1_	141.0	84.5	29.2	55.3
TiZrNbCr	163.9	100.7	27.0	73.7
TiZrNbCr_0.4_	176.4	114.6	16.0	98.6

### 3.3. Elastic Modulus

**Figure 2:**
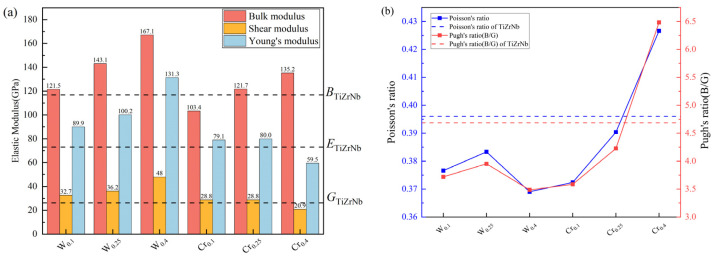
(**a**) The elastic modulus of TiZrNbX is obtained via the VRH approximation. (**b**) The Pugh’s ratio and Poisson’s ratio of TiZrNbX.

The elastic modulus is one of the key mechanical properties of materials. The calculated values from this work and previous research studies on two equiatomic RHEAs are shown in Table 3. As shown in Table 3, the calculated values exhibit significant deviations from values based on the CPA method. This is probably due to the fact that the CPA method ignores the influence of the local environment on the overall mechanical properties. The calculated values were basically the same compared to previous SQS methods in this work. This suggests that supercell size does not significantly affect the calculated mechanical property values. In addition, Figure 2a presents the bulk modulus, shear modulus and Young’s modulus of TiZrNbX and TiZrNb alloys obtained by Voigt–Reuss–Hill approximations [37]. According to Figure 2a, the doping of W enhances the elastic modulus values of TiZrNb alloys in all aspects. Thus, the resistance of the material to various types of deformation has been enhanced. Furthermore, this enhancement effect increases with the increase in W concentration. The influence of Cr on TiZrNb alloys is multifaceted. In summary, Cr decreases the shear modulus and Young’s modulus of TiZrNb, thereby weakening the shear and elastic deformation resistance of TiZrNb alloys. The influence on the bulk modulus depends on the Cr level. Although low concentrations of Cr have a weakening effect, high concentrations of Cr will enhance the bulk modulus.

The bulk modulus and shear modulus are the resistance of material to different types of deformation. Thus, the competition between them is related to the ductility of the material. Poisson’s ratio is also commonly associated with the ductility of materials [15]. For polycrystalline materials, the Pugh ratio (B/G > 1.75) and Poisson’s ratio (v>0.26) show ductility and the inverse for brittleness [38]. The higher the Pugh ratio, the better the material’s ductility; conversely, the lower the Pugh ratio, the more brittle the material. Figure 2b shows the Pugh’s ratio and Poisson’s ratio of the RHEAs. It is clear that all the alloys are relatively ductile. The addition of W and Cr reduced the ductility of the TiZrNb alloys. Although low concentrations of W exhibited a tendency towards improved ductility, increased concentrations of W caused the ductility to decrease again. The findings of this work are consistent with experimental measurements. The addition of W will reduce the ductility of the TiZrNb alloy [39]. However, the ductility of TiZrNbW alloys could be improved to a certain extent by adding an appropriate percentage of W. Furthermore, although Cr alloying can enhance the alloy’s ductility, only high concentrations of Cr have the potential to improve the ductility of TiZrNb alloys. Previous studies have shown that ductile fracture has been observed in the disordered solid solution phase of TiZrNbCr, which was consistent with our calculated value [33]. However, Cr easily forms Laves phases with other elements. These Laves phases lead to stress concentration during loading. Finally, this causes catastrophic fracture.

**Figure 3:**
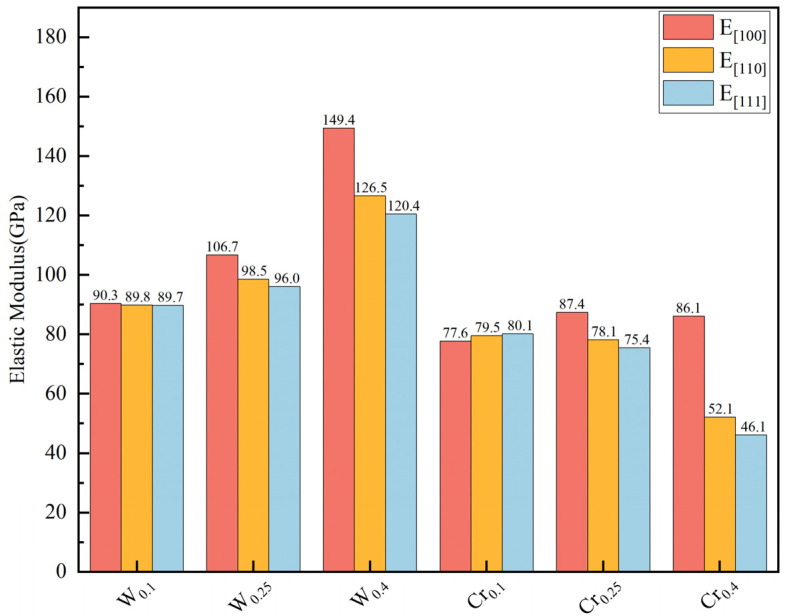
Young’s modulus of RHEAs for different crystal direction.

**Figure 4:**
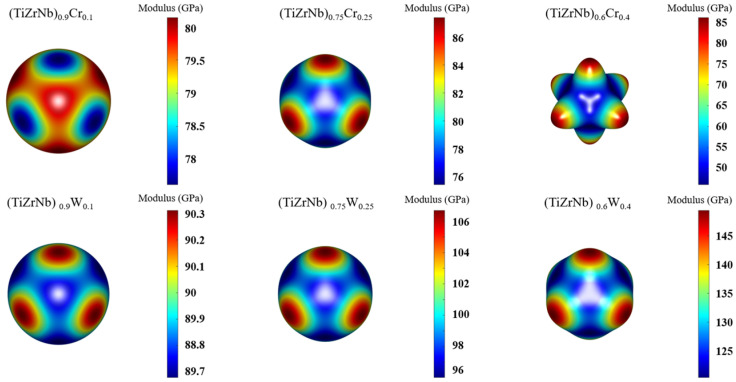
Three-dimensional surface of Young’s modulus for RHEAs.

To further explore the mechanical properties of RHEAs, the Young’s modulus was calculated for the <100>, <011> and <111> directions, respectively [40]. Herein, as shown in Figure 3, it can be seen that the Young’s modulus varies in different directions, indicating that the alloys exhibit anisotropy. From Figure 3, W doping significantly enhances deformation resistance in both directions. The strengthening effect increases with the rising W concentration. However, it is only at low Cr doping levels that the deformation resistance of <100> exhibits a slight improvement. For the <011> and <111> directions, Cr continuously decreases the material’s deformation resistance. To more directly display the anisotropy of the alloy’s Young’s modulus, this work presented a three-dimensional surface of the alloy’s Young’s modulus [41], as shown in Figure 4. When the Young’s modulus exhibits isotropy, the 3D surface exhibits a typical spherical shape. It can be observed that the structure exhibits a near-spherical morphology, only at low levels of W and Cr. As the levels of doping elements increase, the anisotropy of RHEAs gradually increases. The influence of Cr on alloy anisotropy is significantly stronger than that of W.

(2) $$E_{001}=\frac{\left(C_{11}-C_{12}\right)\left(C_{11}+2C_{12}\right)}{C_{11}+C_{12}}$$

(3) $$E_{011}={\left\{\frac{C_{11}+C_{12}}{\left(C_{11}-C_{12}\right)\left(C_{11}+2C_{12}\right)}+\frac{1}{4}\left(\frac{1}{C_{44}}-\frac{2}{C_{11}-C_{12}}\right)\right\}}^{-1}$$

(4) $$E_{111}={\left\{\frac{C_{11}+C_{12}}{\left(C_{11}-C_{12}\right)\left(C_{11}+2C_{12}\right)}+\frac{1}{3}\left(\frac{1}{C_{44}}-\frac{2}{C_{11}-C_{12}}\right)\right\}}^{-1}$$

### 3.4. Theoretical Strength

The yield strength of RHEAs originates from the intrinsic lattice friction to the dislocation motion and the additional strength provided by other strengthening mechanisms such as solution strengthening ($\Delta \sigma_{SSS}$), grain boundary strengthening ($\Delta \sigma_{gb}$), precipitation strengthening ($\Delta \sigma_{ppt}$), and dislocation strengthening ($\Delta \sigma_{\rho i}$), etc. Therefore, the overall yield strength of RHEAs is expressed as follows [42]:

(5) $$\sigma_{y}=\sigma_{y}^{mix}+\Delta \sigma_{SSS}+\Delta \sigma_{gb}+\Delta \sigma_{ppt}+\Delta \sigma_{\rho i}+\cdots$$

where $\sigma_{y}^{mix}$ is defined as $\sigma_{y}^{mix}=\sum_{i=1}^{n}c_{i}\sigma_{y_{i}}$.$\sigma_{y_{i}}$ is the yield strength of metal, as shown in Table 4. Previous studies found that the proportion of solid solution strengthening in HEAs was two to three times that in conventional alloys [5]. Similarly, it was also found that the strengthening effects other than solution strengthening had little contribution to the yield strength in the RHEAs, and thus can be ignored. The yield strength of BCC RHEAs can be expressed as follows:

(6) $$\sigma_{y}^{cal}=\sigma_{y}^{mix}+\Delta \sigma_{SSS}$$

The solid solution strengthening effect will be estimated based on the Senkov approach. In the Senkov approach, the atomic radius mismatch ($\delta_{r_{i}}$) and shear elastic modulus mismatch ($\delta_{G_{i}}$) of HEAs are defined as follows [43]:

(7) $$\delta_{r_{i}}=\frac{9}{8}\sum_{i\neq j,j=1}^{n}c_{j}\delta_{r_{ij}}$$

(8) $$\delta_{G_{i}}=\frac{9}{8}\sum_{i\neq j,j=1}^{n}c_{j}\delta_{G_{ij}}$$

where $c_{j}$ is the atomic percentage of the j element in the alloy. $\delta_{G_{ij}}$ and $\delta_{r_{ij}}$ represent the atomic radius mismatch and shear modulus mismatch between atoms i and j, respectively. Table 4 presents the atomic radii and shear moduli of pure metals. $\delta_{G_{ij}}$ and $\delta_{r_{ij}}$ are calculated as follows:

(9) $$\delta_{G_{ij}}=2\frac{G_{i}-G_{j}}{G_{i}+G_{j}}$$

(10) $$\delta_{r_{ij}}=2\frac{r_{i}-r_{j}}{r_{i}+r_{j}}$$

Based on the Gypen method [44], the contribution of each element to the solid solution strengthening effect is as follows:

(11) $$\Delta \sigma_{SSS}={\left(\sum_{i=1}^{n}\Delta \sigma_{i}^{\frac{3}{2}}\right)}^{\frac{2}{3}}$$

here, $\Delta \sigma_{i}$ represents the incremental solid solution strengthening effect due to element i, which can be calculated as follows:

(12) $$\Delta \sigma_{i}=AGf_{i}^{\frac{4}{3}}c_{i}^{\frac{2}{3}}$$

where A is the dimensionless constant and A=0.04 [6,45]. The G is the shear modulus of the alloy. $f_{i}$ is the coupling parameter for the RHEAs atomic radius mismatch and shear modulus mismatch. At present, the solid solution strengthening theory does not provide a uniform method for coupling these two effects. Therefore, the researchers have attempted a series of formulas to obtain the best fit with their experimental measurements. This work uses the formula suggested by Kostorz and Mihailovich [44]:

(13) $$\varepsilon =\sqrt{\delta \mu_{ave}^{2}+\left(\alpha \delta r_{ave}^{2}\right)}$$

where α is dependent on edge dislocation or screw dislocation. Past research [45,46] found that setting the α value to nine is a relatively appropriate choice.

(14) $$\delta_{ij}^{atom}=\frac{1}{N_{fs}}\sum_{j=1}^{N_{fs}}2\frac{r_{i}-r_{j}}{r+r_{j}}$$

(15) $$G_{ij}^{atom}=\frac{1}{N_{fs}}\sum_{j=1}^{N_{fs}}2\frac{G_{i}-G_{j}}{G_{i}+G_{j}}$$

The approach proposed by Senkov is based on the Labusch model [47]. In the Labusch model, only the interaction between the solute and the solvent is considered. This apparently does not apply to high entropy alloys. Although the Senkov approach breaks this assumption, the mismatch caused by atomic pairs is dependent on the local environment. The approach only estimates the strength of HEAs by integrating all elements. This may also be one of the reasons for the elemental discrepancy in its estimated accuracy. Previous research has indicated that this approach has a minor estimation error for TaNbHfZrTi [43]. However, the estimation results for HEAs involving W and Mo have been less than satisfactory [45]. The SQS could effectively simulate the local atomic environment of materials at the atomic scale. Therefore, this work will attempt to capture the mismatches from the local atomic environment of each atom within the SQS.

**Figure 5:**
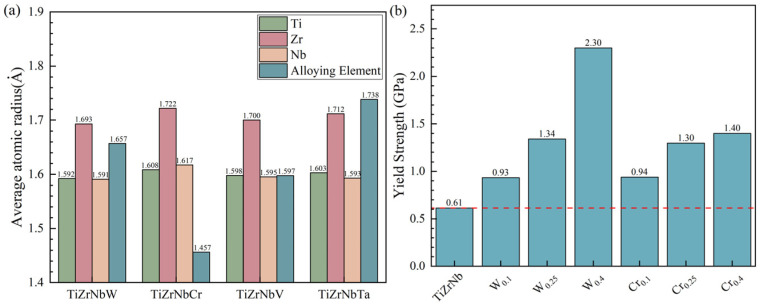
(**a**) The average radius of each atom in the equiatomic TiZrNbX RHEAs by Voronoi tessellation method. (**b**) The yield strength of TiZrNbX RHEAs is estimated using a modified Senkov model.

This work attempts to calculate the average lattice distortion by taking into account the influence of the first neighbors atoms in the crystal. This method is achieved using a Perl script. The steps are as follows: Firstly, utilize a Perl script to scan the types of the central atom and its nearest neighbors. Secondly, the atomic radius mismatch and shear mismatch caused by the central atom are corrected using Equations (14) and (15). Finally, the lattice distortion caused by a specific type of atom is represented by the average mismatch of all atoms of that same type. Then, the Voronoi tessellation method is used to acquire local atomic radii [48]. Figure 5a presents the average radius of each atom calculated using the Voronoi tessellation method. Table 5 displays the estimated yield strength values for four TiZrNbX alloys, based on the modified Senkov method. The values for TiZrNbV and TiZrNbTa were from previous studies [46]. As shown in Table 5, the modified Senkov approach with radius values from the hard sphere model provides estimates closer to the experimental values than those from the Senkov approach for TiZrNbW and TiZrNbCr. For TiZrNbV and TiZrNbTa, both methods supply satisfactory estimated values (error < 10%). However, the adoption of the modified Senkov approach that accounts for local atomic radii instead leads to further deviation from the actual values. This work will attempt to explain this phenomenon. The atomic radius in the Voronoi tessellation method is derived from the equivalent spheres formed by dividing the surrounding space into regions centered on the atom. In practice, the charge transfer effect between elements within an alloy influences the atomic radii.

The Voronoi tessellation method is based on a three-dimensional spatial geometric topological segmentation approach centered on atomic centers. Thus, it partitions cells solely through the spatial coordinates of atoms. As such, it does not involve physical information such as the electronic structure of atoms and interatomic interactions. For an ideal crystal, the Voronoi polyhedral was constructed with atomic centers as the core achieving uniform spatial partitioning, with identical equivalent radii for all cells. Even after structural relaxation, the deviation of atoms from their ideal lattice sites only induces minor fluctuations in the equivalent radii, which ultimately leads to an underestimation of the atomic radius mismatch of the system. The atomic radius mismatch acquired through the two methods is shown in Table 6, where $\bar{\delta_{r_{Ti}}}$ and $\bar{\delta_{r_{X}}}$ represent the sum of the atomic radius mismatch between the matrix element and the sum of the radius mismatch of the doping element atom, respectively. As shown in Table 6, the atomic radius mismatch calculated by the Voronoi tessellation method is significantly lower than that calculated by the hard sphere model. Finally, the differing precision displayed by various alloys is due to their distinct origins of strengthening. Solid solution strengthening primarily depends on shear modulus mismatch and atomic radius mismatch. The purposes of doping are to provide a greater mismatch and to enhance the strength of materials. Therefore, the doping of V will only create a larger atomic radius mismatch. W and Ta doping primarily induce a larger shear modulus mismatch (Cr simultaneously enhances both effects). However, the atomic radius mismatch was estimated to be too small, causing the largest deviation in TiZrNbV. For TiZrNbW RHEAs, the yield strength primarily originates from the shear modulus mismatch, which leads to a relatively small deviation of the estimates.

Figure 5b presents the modified Senkov approach strength estimation values for TiZrNbW and TiZrNbCr RHEAs, using the hard sphere model. As shown in Figure 5b, the alloying of W or Cr enhances the strength of RHEAs. Moreover, the strengthening effects were increasing as the doping composition increased. The strengthening effect of W was higher than Cr as the doping composition increased. One reason for this phenomenon is the electron cloud rearrangement effect induced by heavy elements. Specifically, the yield strength of RHEAs exhibits a power–law relationship with the electron work function [49]. The increasing W content leads to an increase in the electron work function of the alloy, thereby improving the strength of the material. It should be clearly noted that no systematic tests are performed in this work to identify the maximum doping content threshold of the modified Senkov approach. Thus, the precise applicable composition boundary of the model cannot be definitively determined at this stage. Subsequent investigations coupled with high-throughput experiments can be carried out to further define the applicable scope and upper content limit of the model.

**Table 5:** Experimental yield strength and theoretical values for the TiZrNbX RHEAs.

Alloy	$\sigma_{y}^{\exp}$	$\sigma_{y}^{senkov}$	$\sigma_{y}^{senkov_{op}}$	$\sigma_{y}^{senkov_{opv}}$
TiZrNbW	1305.3 [39,50,51]	1571.0	1339.8	1056.34
TiZrNbCr	1260.0 [33]	1476.0	1296.3	947.4
TiZrNbV	1009 [52]	1032	915.0	439.7
TiZrNbTa	1100 [53]	1016	1040.2	681.9

Note: $\sigma_{y}^{senkov_{op}}$ is the theoretical value obtained by using the modified Senkov approach and the hard-sphere model atomic radius. $\sigma_{y}^{senkov_{opv}}$ is the theoretical value obtained by using the modified Senkov approach and Voronoi tessellation method atomic radius.

**Table 6:** RHEAs hard sphere model and Voronoi tessellation method atomic radius mismatch ($\bar{\delta_{r_{ij}}}=\left|\left(r_{i}-r_{j}\right)/\left(r_{i}+r_{j}\right)\right|$).

	Method	$\bar{\delta_{r_{TiZr}}}$	$\bar{\delta_{r_{TiNb}}}$	$\bar{\delta_{r_{ZrNb}}}$	$\bar{\delta_{r_{Ti}}}$	$\bar{\delta_{r_{XTi}}}$	$\bar{\delta_{r_{XZr}}}$	$\bar{\delta_{r_{XNb}}}$	$\bar{\delta_{r_{X}}}$
TiZrNbW	hard sphere	4.97%	1.14%	6.11%	12.22%	3.36%	8.32%	2.22%	13.89%
	VTM	3.08%	0.04%	3.12%	6.24%	2.01%	1.08%	2.04%	5.13%
TiZrNbCr	hard sphere	4.97%	1.14%	6.11%	12.22%	7.86%	12.78%	6.72%	27.36%
	VTM	3.41%	0.28%	3.13%	6.82%	4.95%	8.34%	5.22%	18.51%
TiZrNbV	hard sphere	4.97%	1.14%	6.11%	12.22%	5.26%	10.20%	4.12%	19.57%
	VTM	3.10%	0.08%	3.18%	6.36%	0.01%	3.12%	0.07%	3.19%
TiZrNbTa	hard sphere	4.97%	1.14%	6.11%	12.22%	1.11%	6.08%	0.03%	7.22%
	VTM	3.28%	0.31%	3.59%	7.18%	4.05%	0.77%	4.36%	9.18%

Note: VTM is Voronoi tessellation method.

### 3.5. Electronic Properties

**Figure 6:**
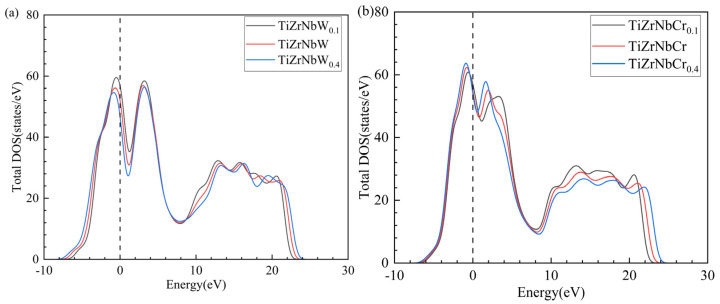
Total density of states for TiZrNbX RHEAs. (**a**)TiZrNbW; (**b**) TiZrNbCr.

Figure 6 shows the total electronic density of states for RHEAs. Since electrons near the Fermi level have relatively important effects on material properties, this work only analyzed the electronic region ranging from −10 eV to 30 eV. As shown in Figure 6, the peak in the density of states (>0) observed near the Fermi level for all the RHEAs indicates that these crystals exhibit metallic behaviors. Secondly, the total density of states for TiZrNbX RHEAs exhibit no significant differences, demonstrating that the BCC remains stable after Cr and W doping. Then, Table 7 provides the Fermi level and pseudogap of the TiZrNbX alloy. A lower Fermi level suggests a more stable crystal structure [54,55]. It can be observed that the Fermi level of these alloys decreases with an increasing dopant concentration, confirming that the BCC becomes more stable. This is consistent with the enhanced stability of the BCC structure, which is attributed to the increased proportion of the BCC phase. The pseudogap also reflects the strength of the covalent property within the RHEAs. The stronger the covalent property, the weaker the ductility of the material. W doping causes the pseudogap to first decrease and then increase with an increasing W concentration. The doping of Cr causes the pseudogap to decrease continuously. It is proof that Cr and an appropriate amount of W improve the ductility of the material. This corresponds to the former analysis of the B/G ratio and Poisson’s ratio.

**Figure 7:**
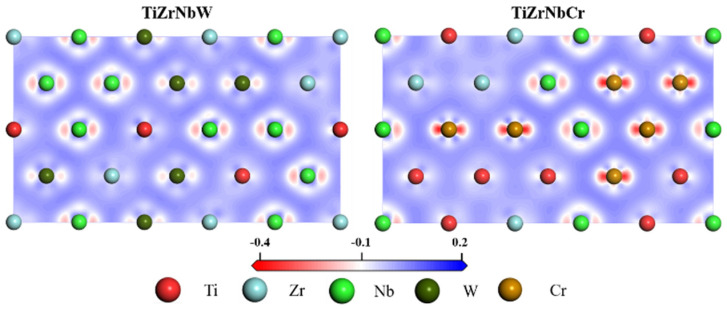
Electronic density difference in TiZrNbW and TiZrNbCr along the crystal plane (110).

Figure 7 shows the electronic density difference (EDD) along the (110) plane for the two equiatomics, TiZrNbCr and TiZrNbW. For these two alloy systems, the electron clouds surrounding Ti and Zr atoms are primarily circular, with weak directionality. There is little directed overlap between these electron clouds, which is typical of metallic properties [56]. In contrast, Nb, W and Cr display an electron cloud overlap with the surrounding atoms, indicating strongly directional electron interactions. This reflects the covalent properties between atoms. This also indirectly proves that the alloying of W and Cr enhances the local covalency and improves the macroscopic strength of the materials.

This work was only focused on the mechanical properties of solid solutions formed after W and Cr alloying. Nevertheless, the addition of W and Cr is prone to induce the formation of intermetallic phases and segregation in actual manufacturing processes. Therefore, it is still necessary to explore homogenization techniques for this type of alloy. Secondly, although this work was extended to calculate the mismatch for atomic local environments, it did not consider potential short-range reinforcement because the atomic arrangement is entirely random in the SQS. On the other hand, while the Voronoi tessellation method supplemented the varying atomic radii in the local environment, this method lacks inherent physical information. These factors will eventually influence lattice distortion. Finally, we have provided only a qualitative explanation for the reasons behind the reinforcement of the W element. This work does not offer any relevant quantitative analysis. In the next steps, the team will explore these aspects in greater detail.

## 4. Conclusions

This work investigated the influence of W and Cr on the mechanical properties of TiZrNb alloys through SQS and first principles. Firstly, W and Cr led to a decrease in the lattice constant of the TiZrNb alloys. The calculated values of the elastic modulus and yield strength for the equiatomic TiZrNbX alloy agreed with previous studies. W alloying improved the resistance to all forms of deformation. However, Cr only enhanced the resistance to bulk deformation. The addition of W and Cr weakens the ductility of TiZrNb alloys to a certain extent. Although high concentrations of Cr or low concentrations of W might slightly improve the plasticity, the risk of brittle fracture due to the formation of intermetallic phases between W and Cr and the matrix elements required attention. Furthermore, W and Cr were unfavorable for the isotropy of the mechanical properties. Cr caused more severe damage to isotropy. Subsequently, this work proposed the Senkov approach that considered local atomic environments. This approach enabled improved estimation accuracy for the yield strength of TiZrNbW and TiZrNbCr. Theoretical calculations indicated that W exhibited a stronger solid solution strengthening effect than Cr. Electronic property analysis found that W and Cr could lower the Fermi level to enhance BCC stability. The covalent properties displayed by W and Cr with surrounding atoms contributed to the strength enhancement. Future work will provide further theoretical guidance for the experiment.

## Figures and Tables

**Table 1:** Lattice constant of TiZrNbX alloy.

Alloy	$a_{\mathrm{Vegard}}$	$a_{SQS}$
TiZrNb	3.377/3.400 * [26]	3.390
TiZrNbW_0.1_	3.356	3.361
TiZrNbW	3.324/3.313 ** [18]	3.320
TiZrNbW_0.4_	3.292	3.283
TiZrNbCr_0.1_	3.330	3.341
TiZrNbCr	3.260	3.263
TiZrNbCr_0.4_	3.190	3.180

Note: * is experimental values and ** is calculated values.

**Table 3:** The comparison of elastic modulus of equiatomic alloys under different methods.

Alloy	B (GPa)	G (GPa)	E (GPa)	Method
TiZrNbW	143.1	36.2	100.2	This work
	151.1	31.6	88.7	SQS [37]
	143.5	62.9	164.5	CPA [18]
TiZrNbCr	121.7	28.8	80.0	This work
	134.2	27.2	76.6	SQS [37]

Note: B is bulk modulus. G is shear modulus. E is Young’s modulus.

**Table 4:** Physical parameters of pure metal.

Properties	Ti	Zr	Nb	W	Cr
Atomic radius ($\overset{\circ }{A}$)	146.2	161.5	142.9	136.7	124.9
Shear modulus (GPa)	44	33	38	161	115
Yield strength (MPa)	195	280	240	550	140

**Table 7:** Fermi level and pseudogap of TiZrNbX RHEAs.

Alloy	Fermi Level (eV)	Pseudogap (eV)
TiZrNbW_0.1_	56.71	3.71
TiZrNbW	52.02	3.65
TiZrNbW_0.4_	46.65	4.03
TiZrNbCr_0.1_	57.02	3.90
TiZrNbCr	54.96	2.76
TiZrNbCr_0.4_	54.03	2.52

## Data Availability

The original contributions presented in this study are included in the article. Further inquiries can be directed to the corresponding authors.
